# Generational trends in reproductive factors among women in the US: implications for breast cancer incidence

**DOI:** 10.1186/s13058-026-02222-x

**Published:** 2026-02-03

**Authors:** Lin Yang, Adetunji T. Toriola

**Affiliations:** 1 Department of Cancer Epidemiology and Prevention Research, Cancer Care Alberta, Calgary, AB Canada; 2 Departments of Oncology and Community Health Sciences, Cumming School of Medicine, University of Calgary, Calgary, AB Canada; 3 Division of Public Health Sciences, Department of Surgery, Washington University School of Medicine, 660 S Euclid Ave, Campus Box 8100, St. Louis, MO 63110 USA; 4 Alvin J. Siteman Cancer Center, Washington University School of Medicine, St. Louis, MO USA; 5 Arthur Child Cancer Centre, 7th Floor, Office YC071433, 3395 Hospital Drive N.W, Calgary, AB T2N 5G2 Canada

**Keywords:** Breast cancer risk, Reproductive factors, Generational trends, Birth cohort, Age at menarche, Parity

## Abstract

**Background:**

Reproductive factors are key breast cancer risk factors, yet contemporary generational patterns remain unclear. We aimed to evaluate trends in reproductive factors among US women born between 1910 and 2000.

**Methods:**

We conducted a serial, cross-sectional analysis of reproductive factors using data from the US National Health and Nutrition Examination Survey (NHANES) collected between 1999 and 2020. Female participants were grouped by birth cohorts (1910–1930, then by 10-year to 2000). Self-reported data were used to derive age at menarche, prevalence of age at menarche < 12 years, age at natural menopausal, prevalence of first live birth after age 30, and lifetime number of live births.

**Results:**

Data on 28,481 US women were analyzed. From birth cohort 1910–1930 to 1990–2000, the mean age at menarche declined from 13.0 (95%CI 12.9 to 13.1) to 12.4 (95%CI 12.3 to 12.5) years (difference = − 0.6, 95%CI − 0.7 to − 0.5, *p* for trend < 0.001). This decline was observed in both US (12.9 [95%CI 12.9 to 13.0] to 12.4 [95%CI 12.3 to 12.5]) and non-US born (13.3 [95%CI 13.1 to 13.6] to 12.3 [95%CI 12.1 to 12.5]) women. From birth cohort 1910–1930 to 1990–2000, the prevalence of age at menarche < 12 years increased from 14.2% (95%CI 12.4 to 16.1) to 26.5% (95%CI 24.3 to 28.9%). The prevalence of first live birth after age 30 increased from 4.7% (95%CI 3.1 to 6.9%) to 10.6% (95%CI 7.3 to 15.0%) from birth cohort 1910–1930 to 1980–1990. Among women who attained menopause (up to birth cohort 1950–1960), no significant changes were observed for age at natural menopausal. However, the average lifetime number of live births declined from 3.5 to 2.4 with a significant decline in the proportion of grand multiparous women.

**Conclusions:**

Over the past century, women in the US are attaining menarche earlier, more likely to have their first birth after age 30 and having fewer births. Parsing the extent to which changes in reproductive factors are contributing to the rising incidence of estrogen receptor positive breast cancer, especially in premenopausal women, is necessary to devise long-lasting and sustainable preventive strategies.

**Supplementary Information:**

The online version contains supplementary material available at 10.1186/s13058-026-02222-x.

## Background

Breast cancer incidence is increasing at an annual rate of approximately 1% in the US across all racial and ethnic groups [1]. Notably, this rise is more pronounced in younger women aged 20–49 years compared to those over 50 [2]. The most dramatic shifts in breast cancer risk are observed across birth cohorts, with women born in early 1990s experiencing a 25% higher risk compared to those born in late 1950s [2]. This increase is largely driven by estrogen receptor-positive subtypes (ER+/PR+ and ER+/PR−), while estrogen receptor-negative subtypes (ER−/PR+ and ER−/PR−) have declined [2].

ER+ breast cancer represents the majority of cases and are influenced by adiposity and reproductive factors, though these associations differ by menopausal status [3–7]. While adiposity can explain some of the rise in incidence of postmenopausal breast cancer, it does not account for the increasing incidence in premenopausal women, where higher adiposity is inversely associated with risk [8]. The associations of reproductive factors such as ages at menarche and menopause, parity with breast cancer underscore the influence of sex steroid hormones on breast cancer development, particularly ER+ tumors [7]. A one-year earlier menarche is associated with a 7% higher risk of breast cancer in women under 45 and a 4% increase in women over 65 [9].

While previous studies have documented temporal changes in individual reproductive factors [10–13], few have adopted a comprehensive approach across multiple reproductive factors, nor have they systematically examined these patterns by birth cohort. Moreover, few have evaluated how these trends differ across several factors including race/ethnicity and country of birth, limiting our understanding of how reproductive factors have evolved in relation to the growing breast cancer burden. To address these gaps, this serial cross-sectional study aims to comprehensively describe the historical trends in, and current patterns of, reproductive factors among US women born between 1910 and 2000.

## Methods

### Study population

The National Health and Nutrition Examination Survey (NHANES) was designed to provide nationally representative estimates of the prevalence of health, nutrition, and potential risk factors in the US population [14]. Since 1999, the NHANES study has continuously surveyed a nationally representative, complex, stratified, multistage probability sample of the civilian non-institutionalized US population in 2-year cycles. We included women ≥ 16 years who reported data on reproductive factors in the reproductive health questions from the 1999–2000 and 2017-March 2020 pre-pandemic survey cycles. Data on age, race/ethnicity (Hispanic, non-Hispanic black, non-Hispanic white, Remaining) and country of birth (US and non-US born) were derived from sociodemographic questions.

### Reproductive factors

Women were asked about their age when first menstrual period occurred. Age at menarche was defined as the age at first menstrual cycle between 9 and 16 years, to exclude women with potentially underlying pathological cause of delayed menarche. Additional categorical data for age at menarche were created for < 10 versus  ≥ 10 years, < 11 versus  ≥ 11 years, < 12 versus  ≥ 12 years, < 13 versus  ≥ 13 years. Next, women were asked if they had regular periods in past 12 months (Yes/No) and the reasons for not having regular periods (pregnancy, breast feeding, hysterectomy, menopause, medical conditions-treatments, etc.). Women were also asked about age at last menstrual period. Age at menopause was defined as the age at last menstrual period between 40 and 62 years to exclude women with potentially underlying pathological cause of premature or late onset menopause. To determine natural menopause, women were further asked if they had a hysterectomy (Yes/No), and if they had both ovaries removed (Yes/No). Women who responded “menopause” as the reason for not having regular period in past 12 months, and who did not undergo a hysterectomy prior to their last menstrual period were classified as natural menopause. Reproductive lifespan was derived by subtracting age at menarche from age at menopause.

With respect to parity, women were asked about their age at first live birth, age at last live birth, and the number of live births. Information on parity were derived for first live birth within 10 years from menarche among women > 26 years, and for first live births at age > 30 among women > 30 years. Among women who had attained menopause, number of live births were summarized and categorized into 0 (nulliparous), 1–2, 3–4 and 5 + (grand multiparous).

### Birth cohort

NHANES does not record participants’ year of birth. Using demographic variables and study cycle, year of birth was derived as the the lower bound of study cycle minus participants' age (i.e., the estimated year of birth for a participant age 40 in study cycle 1999–2000 would be 1959).

Age at menarche was analyzed among women who were at least 16 years at the time of survey, spanning eight birth cohorts: 1910–< 1930, 1930–< 1940, 1940–< 1950, 1950–< 1960, 1960–< 1970, 1970–< 1980, 1980–< 1990, and 1990–< 2000. Age at menopause and reproductive lifespan were analyzed among women who were at least 62 years at the time of survey, spanning four birth cohorts: 1910–< 1930, 1930–< 1940, 1940–< 1950, and 1950–< 1960.

The proportion of women who had first live birth within 10 years from menarche and first live birth at age > 30 were analyzed among seven birth cohorts: 1910–< 1930, 1930–< 1940, 1940–< 1950, 1950–< 1960, 1960–< 1970, 1970–< 1980, 1980–< 1990. In addition, number of lifetime live births were analyzed among women who had attained menopause across four birth cohorts: 1910–< 1930, 1930–< 1940, 1940–< 1950, and 1950–< 1960.

### Statistical analysis

All statistical analyses were performed using STATA version 17.0 (STATA Corp., College Station, Texas). Survey analysis procedures were used to account for the sample weights, stratification, and clustering of the complex sampling design to ensure nationally representative estimates [15]. Sample sizes were estimated overall and by birth cohort, race/ethnicity, and country of birth. We calculated weighted means and 95% confidence intervals (95%CIs) for age at menarche, age at menopause and reproductive lifespan overall and by birth cohort, race/ethnicity, and country of birth. We calculated weighted prevalence and 95%CI for age at menarche < 10, < 11, < 12, and < 13 years overall and the weighted prevalence and 95%CI for age at menarche < 12 years by race/ethnicity and birth cohort. For parity, we calculated weighted prevalence and 95%CI for women who had first live birth within 10 years of menarche and first live birth at age > 30 overall and by birth cohort, race/ethnicity, and country of birth. The means and 95%CI of number of live births and distribution were estimated by birth cohort. Trends in reproductive factors over time were evaluated using birth cohort as a continuous variable as well as including interaction terms with race/ethnicity and country of birth, respectively, in multivariable regressions. Sensitivity analyses were performed by using the upper bound of study cycle to derive year of birth (i.e., the estimated year of birth for a participant age 40 in study cycle 1999–2000 would be 1960). All statistical tests were two-sided, and statistical significance was set at *P* < 0.05.

## Results

Data on 28,481 women were analyzed. Sample sizes from the first (1910–< 1930) through last (1990–< 2000) birth cohort are shown in eTable 1. Most participants were non-Hispanic White (68.4%) and born in the US (85.2%).

### Age at menarche

**Fig. 1:**
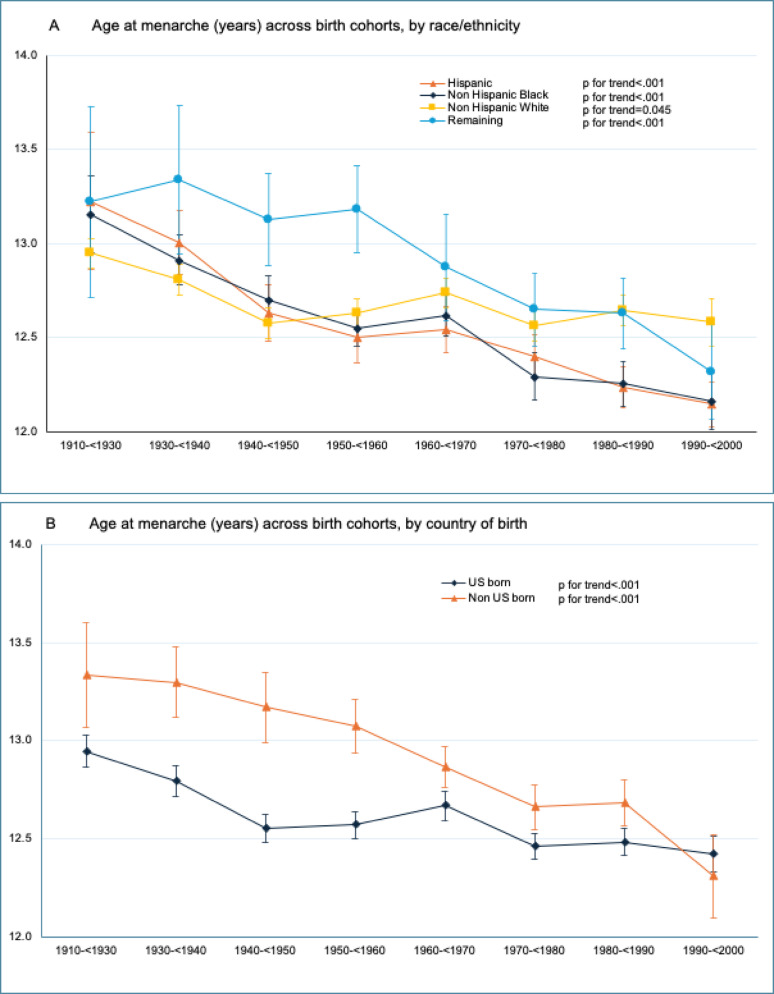

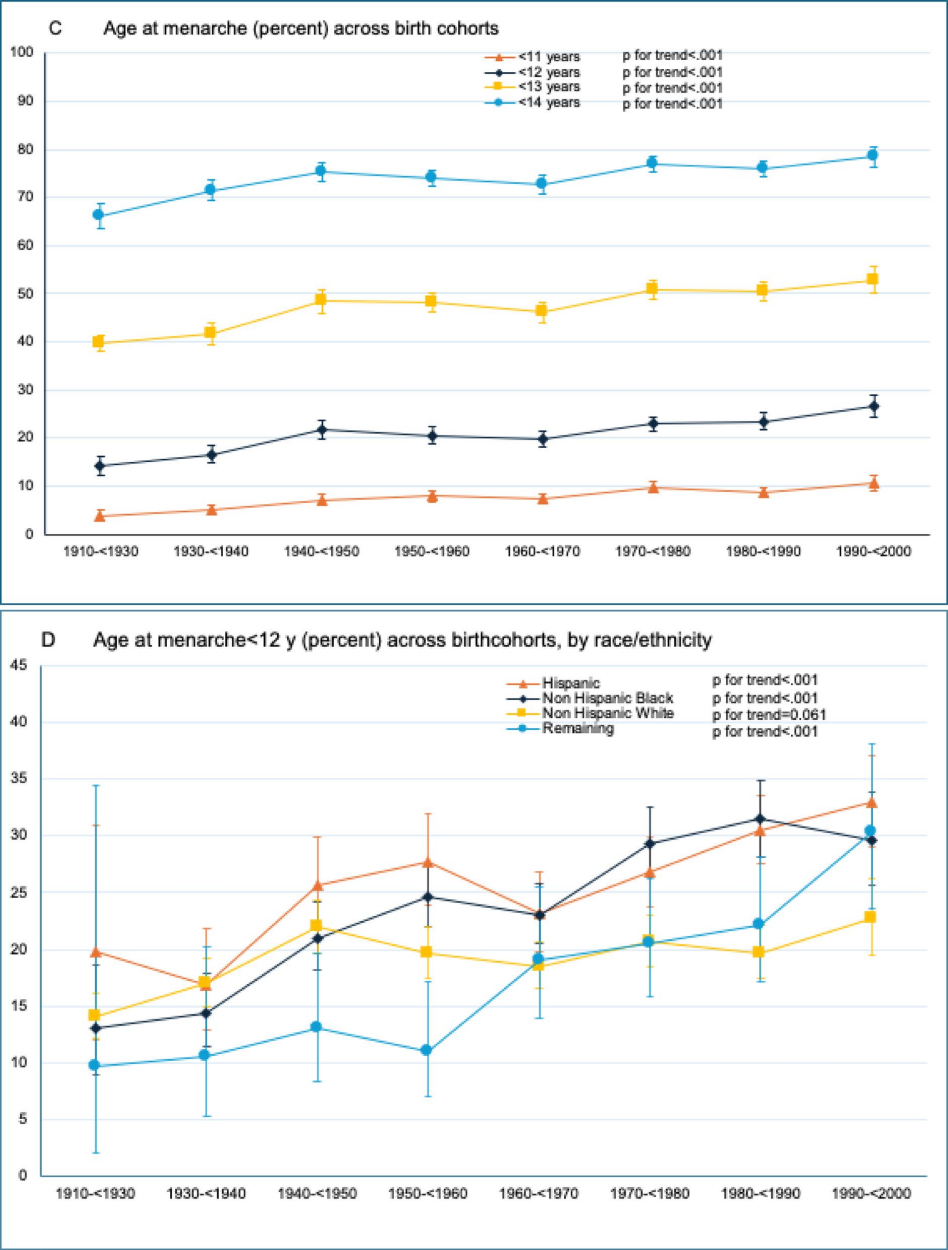
**A**–**D** Weighted trends in age at menarche among US women in 1910–< 1930 through 1990–2000 birth cohorts. Trends in age at menarche across birth cohorts were evaluated using *P* values for trend by modeling birth cohort as a continuous variable in univariate regression models. Data are from the National Health and Nutrition Examination Survey and were weighted to be nationally representative. Error bars indicate 95% CIs

The mean age at menarche declined from 13.0 (95%CI 12.9 to 13.1) in the 1910–< 1930 birth cohort to 12.4 (95%CI 12.3 to 12.5) years (difference = − 0.6, 95%CI − 0.7 to − 0.5, *p* for trend < 0.001) in the 1990–< 2000 birth cohort (Table 1). The shift to an earlier age at menarche was observed in both US and non-US born women (Fig. 1A, *p* for interaction < 0.001). Nevertheless, non-US born women had a later age at menarche compared to the US born women (13.3 [95%CI 13.1 to 13.6] vs. 12.9 [95%CI 12.9 to 13.0], *p* = 0.009) in the 1910–< 1930 birth cohort. This gap narrowed over time and converged (12.3 [95%CI 12.1 to 12.5] vs. 12.4 [95%CI 12.3 to 12.5], *p* = 0.373) in the 1990–< 2000 birth cohort.

**Table 1:** Weighted trends in mean age at menarche, overall and by race/ethnicity and country of birth among US women birth cohort 1910–< 1930 to 1990–< 2000^a^

	Trends in age at menarche (year, mean [SE]) birth cohort								P for trend^b^	Last vs. 1st birth cohort difference (95% CI)^c^
	1910–< 1930	1930–< 1940	1940–< 1950	1950–< 1960	1960–< 1970	1970–< 1980	1980–< 1990	1990–< 2000		
Overall	13.0	12.8	12.6	12.6	12.7	12.5	12.5	12.4	< 0.001	− 0.6
	(12.9 to 13.1)	(12.8 to 12.9)	(12.6 to 12.7)	(12.6 to 12.7)	(12.6 to 12.8)	(12.4 to 12.6)	(12.5 to 12.6)	(12.3 to 12.5)		(− 0.7 to − 0.5)
Race/ethnicity										
Hispanic	13.2	13.0	12.6	12.5	12.5	12.4	12.2	12.1	< 0.001	− 1.1
	(12.9 to 13.6)	(12.8 to 13.2)	(12.5 to 12.8)	(12.4 to 12.6)	(12.4 to 12.7)	(12.3 to 12.5)	(12.1 to 12.3)	(12.0 to 12.3)		(− 1.5 to − 0.7)
Non-Hispanic Black	13.2	12.9	12.7	12.5	12.6	12.3	12.3	12.2	< 0.001	− 1.0
	(13.0 to 13.4)	(12.8 to 13.1)	(12.6 to 12.8)	(12.5 to 12.7)	(12.5 to 12.7)	(12.2 to 12.4)	(12.1 to 12.4)	(12.0 to 12.3)		(− 1.2 to − 0.7)
Non-Hispanic White	12.9	12.8	12.6	12.6	12.7	12.6	12.6	12.6	0.001	− 0.4
	(12.9 to 13.1)	(12.7 to 12.9)	(12.5 to 12.7)	(12.6 to 12.7)	(12.7 to 12.8)	(12.5 to 12.7)	(12.6 to 12.7)	(12.5 to 12.7)		(− 0.5 to − 0.2)
Remaining^d^	13.2	13.3	13.1	13.2	12.9	12.6	12.6	12.3	< 0.001	− 0.9
	(12.7 to 13.7)	(13.0 to 13.7)	(12.9 to 13.4)	(13.0 to 13.4)	(12.6 to 13.2)	(12.5 to 12.8)	(12.4 to 12.8)	(12.1 to 12.6)		(− 1.5 to 0.3)
Country of birth										
US born	12.9	12.8	12.6	12.6	12.7	12.5	12.5	12.4	< 0.001	− 0.5
	(12.9 to 13.0)	(12.7 to 12.9)	(12.5 to 12.6)	(12.5 to 12.6)	(12.6 to 12.7)	(12.4 to 12.5)	(12.4 to 12.6)	(12.3 to 12.5)		(− 0.6 to − 0.4)
Non-US born	13.3	13.3	13.2	13.1	12.9	12.7	12.7	12.3	< 0.001	− 1.0
	(13.1 to 13.6)	(13.1 to 12.5)	(13.0 to 13.4)	(12.9 to 13.2)	(12.8 to 13.0)	(12.6 to 12.8)	(12.6 to 12.8)	(12.1 to 12.5)		(− 1.4 to − 0.7)

^a^All data are weighted to be US nationally representative. Year of birth was derived as the lower bound of study cycle minus participants' age (i.e., the estimated year of birth for a participant aged 40 years in study cycle 1999–2000 would be 1959)

^b^P for trend were calculated using linear regression that included the National Health and Nutrition Examination Survey (NHANES) estimated birth cohort as a continuous variable

^c^Indicates the absolute change in mean age at menarche between birth cohort 1910–< 1930 and 1990–2000, a decrease corresponds to younger age at menarche

^d^ “Remaining” includes race/ethnicity other than non-Hispanic white, non-Hispanic black, or Hispanic, including multiracial

The shift to an earlier age at menarche was observed among all racial/ethnic groups (Fig. 1B, *p* for interaction < 0.001) with larger declines observed among Hispanic (13.2 [95%CI 12.9 to 13.6] to 12.1 [95%CI 12.0 to 12.3]; difference = − 1.1 [95%CI − 1.5 to − 0.7]) and Non-Hispanic Black (13.2 [95%CI 13.0 to 13.4] to 12.2 [95%CI 12.0 to 12.3]; (difference = − 1.0 [95%CI − 1.2 to − 0.7]) women compared to non-Hispanic White women (12.9 [95%CI 12.9 to 13.1] to 12.6 [95%CI 12.5 to 12.7]; difference = − 0.4 [95%CI − 0.5 to − 0.2]).

In the 1910–< 1930 birth cohort, the proportions of women with mean age at menarche < 11, < 12, < 13, and < 14 years were 3.8%, 14.2%, 39.8% and 66.1%, respectively (eTable 2). These proportions increased to 10.5%, 26.5%, 52.9% and 78.5% among women in the 1990–< 2000 birth cohort (eTable 2, Fig. 1C). From 1910–< 1930 to 1990–< 2000 birth cohort, the proportion of women with age at menarche < 12 years increased by 13.1% (95%CI 2.8 to 23.4%), 16.6 (95%CI 10.4 to 22.9%), and 8.7% (95%CI 4.8 to 12.6%) among Hispanic, non-Hispanic Black, and non-Hispanic White women, respectively (Table 2, Fig. 1D).

**Table 2:** Weighted trends in prevalence of age at menarche < 12 years, overall and by race/ethnicity and country of birth among US women birth cohort 1910–< 1930 to 1990–< 2000^a^

	Trends in age at menarche< 12 years (percentage [SE]) birth cohort								P for trend^b^	Last vs. 1st birth cohort difference (95% CI)^c^
	1910–< 1930	1930–< 1940	1940–< 1950	1950–< 1960	1960–< 1970	1970–< 1980	1980–< 1990	1990–< 2000		
Overall	14.2	16.5	21.7	20.5	19.7	23.0	23.4	26.5	< 0.001	12.3
	(12.4 to 16.1)	(14.8 to 18.3)	(19.8 to 23.6)	(18.8 to 22.2)	(18.1 to 21.4)	(21.5 to 24.5)	(21.7 to 25.3)	(24.3 to 28.9)		(9.3 to 15.3)
Race/ethnicity										
Hispanic	19.8	16.8	25.6	27.8	23.1	26.8	30.5	32.9	< 0.001	13.1
	(12.0 to 30.9)	(12.8 to 21.8)	(21.7 to 30.0)	(23.9 to 32.0)	(19.9 to 26.8)	(23.8 to 30.0)	(27.6 to 33.7)	(29.0 to 37.1)		(2.8 to 23.4)
Non-Hispanic Black	13.0	14.3	21.0	24.6	23.0	29.4	31.5	29.6	< 0.001	16.6
	(8.9 to 18.6)	(11.3 to 17.9)	(18.1 to 24.2)	(22.1 to 27.4)	(20.5 to 25.7)	(26.3 to 32.6)	(28.2 to 34.9)	(25.6 to 34.0)		(10.4 to 22.9)
Non-Hispanic White	14.0	16.9	21.9	19.6	18.5	20.7	19.7	22.7	< 0.001	8.7
	(12.2 to 16.2)	(15.0 to 19.1)	(19.7 to 24.4)	(17.5 to 21.9)	(16.5 to 20.6)	(18.5 to 23.0)	(17.4 to 22.1)	(19.6 to 26.3)		(4.8 to 12.6)
Remaining^d^	9.6	10.5	13.0	11.0	19.1	20.6	22.1	30.4	< 0.001	20.8
	(2.1 to 34.5)	(5.2 to 20.2)	(8.4 to 19.7)	(6.9 to 17.1)	(13.9 to 25.5)	(15.9 to 26.3)	(17.1 to 28.2)	(23.7 to 38.1)		(5.1 to 36.6)
Country of birth										
US born	14.5	17.1	22.3	21.2	20.3	23.1	24.2	26.1	< 0.001	11.7
	(12.5 to 16.7)	(15.3 to 19.2)	(20.3 to 24.4)	(19.3 to 23.3)	(18.5 to 22.2)	(21.4 to 24.9)	(22.2 to 26.3)	(23.6 to 28.8)		(8.3 to 15.0)
Non-US born	11.1	10.8	16.9	15.8	17.0	22.3	19.5	29.1	< 0.001	17.9
	(6.1 to 19.4)	(8.0 to 14.5)	(13.1 to 21.6)	(12.9 to 19.2)	(14.3 to 20.1)	(19.5 to 25.3)	(16.7 to 22.7)	(23.4 to 35.5)		(9.3 to 26.6)

^a^All data are weighted to be US nationally representative. Year of birth was derived as the lower bound of study cycle minus participants' age (i.e., the estimated year of birth for a participant aged 40 years in study cycle 1999–2000 would be 1959)

^b^P for trend were calculated using linear regression that included in the National Health and Nutrition Examination Survey (NHANES) estimated birth cohort as a continuous variable

^c^Indicates the absolute change in prevalence of age at menarche < 12 years between birth cohort 1910–< 1930 and 1990–2000, an increase corresponds to higher prevalence of younger age at menarche

^d^ “Remaining” includes race/ethnicity other than non-Hispanic white, non-Hispanic black, or Hispanic, including multiracial

### Age at natural menopause and reproductive lifespan

The mean age at natural menopause (49.2 [95%CI 48.8 to 49.6] to 49.4 [95%CI 49.1 to 49.7], eTable 3) and reproductive lifespan (36.2 [95%CI 35.9 to 36.6] to 36.8 [95%CI 36.5 to 37.1], eTable 4) did not change between the 1910–< 1930 and 1990–< 2000 cohorts.

### *First live birth within 10 years of menarche and first live birth at age* > *30*

The proportion of women who had their first live birth within 10 years of menarche increased from 48.8% (95%CI 45.9 to 51.8) in the 1910–< 1930 birth cohort to 59.3% (95%CI 56.8 to 61.9) in the 1930–< 1940 birth cohort, and declined to 45.3 (95% CI 41.7 to 49.0) through the 1980–< 1990 birth cohort, primarily driven by the trend in non-Hispanic White women (eTable 5). The proportion of women who had their first live birth at age > 30 increased from 4.7% (95%CI 3.1 to 6.9%) in the 1910–< 1930 birth cohort to 10.6% (95%CI 7.3 to 15.0%) in the 1980–< 1990 birth cohort (Table 3). The increasing proportion of having first birth at age > 30 was only observed among women born in the US (from 4.1% [95%CI 2.5 to 6.6%] to 11.3% [95%CI 7.4 to 16.8%]) but not those born outside the US (from 10.1% [95%CI 5.5 to 17.8%] to 8.4% [95%CI 4.7 to 14.6%]) (*p* for interaction < 0.001). The increase in the proportion of women having their first live birth at age > 30 varied by race/ethnicity (*p* for interaction < 0.001), mainly driven by increases in non-Hispanic White women from 4.6% [95%CI 2.9 to 7.1%] in the 1910–< 1930 birth cohort to 15.7% [95%CI 10.1 to 23.6%], difference = 11.2% [95%CI 4.2 to 18.1]), *p* < 0.001) in the 1980–< 1990 birth cohort. Although an increase was observed among non-Hispanic Black women from the 1910–< 1930 (2.9% [95%CI 0.9 to 8.6%] to 1970–< 1980 birth cohort (5.4% [95%CI 4.0 to 7.2]), there was a decline in the 1980–< 1990 birth cohort (2.9% [95%CI 1.1 to 7.6%]).

**Table 3:** Weighted trends in prevalence of first live birth at age > 30, overall and by race/ethnicity and country of birth among US women birth cohort 1910–< 1930 to 1980–< 1990^a^

	Trends in first live birth at age > 30 (percentage [SE]) birth cohort							P for trend^b^	Last vs. 1st birth cohort difference (95% CI)^c^
	1910–< 1930	1930–< 1940	1940–< 1950	1950–< 1960	1960–< 1970	1970–< 1980	1980–< 1990		
Overall	4.7	2.7	5.0	8.5	10.2	10.0	10.6	< 0.001	5.9
	(3.1 to 6.9)	(2.0 to 3.6)	(3.8 to 6.5)	(6.9 to 10.5)	(8.6 to 12.1)	(7.9 to 12.6)	(7.3 to 15.0)		(1.7 to 10.1)
Race/ethnicity									
Hispanic	7.1	5.9	3.6	6.4	5.1	5.6	4.1	0.864	− 2.9
	(2.3 to 19.4)	(3.0 to 11.6)	(2.4 to 5.4)	(4.0 to 10.1)	(3.8 to 7.0)	(4.0 to 7.7)	(2.0 to 8.3)		(− 11.1 to 5.2)
Non-Hispanic Black	2.9	1.4	1.9	4.1	5.4	5.1	2.9	< 0.001	0.01
	(0.9 to 8.6)	(0.6 to 3.1)	(1.0 to 3.7)	(2.7 to 6.0)	(4.0 to 7.2)	(3.4 to 7.7)	(1.1 to 7.6)		(− 4.3 to 4.4)
Non-Hispanic White	4.6	2.5	5.7	9.2	11.9	12.2	15.7	< 0.001	11.2
	(2.9 to 7.1)	(1.7 to 3.5)	(4.1 to 7.7)	(7.0 to 12.0)	(9.7 to 14.4)	(8.8 to 16.8)	(10.1 to 23.6)		(4.2 to 18.1)
Remaining^d^	7.7	4.6	4.2	13.9	14.9	15.7	9.7	0.002	2.1
	(1.1 to 38.9)	(1.1 to 17.2)	(1.8 to 9.3)	(8.6 to 21.7)	(9.2 to 23.1)	(10.7 to 22.5)	(4.7 to 19.2)		(− 14.1 to 18.3)
Country of birth									
US born	4.1	2.1	4.6	8.2	10.3	10.3	11.3	< 0.001	7.2
	(2.5 to 6.6)	(1.4 to 3.1)	(3.4 to 6.2)	(6.4 to 10.4)	(8.5 to 12.4)	(7.7 to 13.7)	(7.4 to 16.8)		(2.1 to 12.3)
Non-US born	10.1	8.0	7.7	10.0	10.0	9.1	8.4	0.662	− 1.8
	(5.5 to 17.8)	(4.9 to 12.8)	(4.6 to 12.6)	(7.0 to 14.0)	(7.3 to 13.7)	(6.8 to 12.1)	(4.7 to 14.6)		(− 9.5 to 5.9)

^a^ All data are weighted to be US nationally representative. Year of birth was derived as the lower bound of study cycle minus partcipants' age (i.e., the estimated year of birth for a participant aged 40 years in study cycle 1999–2000 would be 1959)

^b^ P for trend were calculated using linear regression that included in the National Health and Nutrition Examination Survey (NHANES) estimated birth cohort as a continuous variable

^c^ Indicates the absolute change in prevalence of first live birth over 30 years between birth cohort 1910–< 1930 and 1980–< 1990, an increase corresponds to higher prevalence of delayed first live birth

^d^ “Remaining” includes race/ethnicity other than non-Hispanic white, non-Hispanic black, or Hispanic, including multiracial

### Number of live births

The mean number of live births among women who had attained menopause declined from 3.5 (95%CI 3.3 to 3.7) in the 1910–< 1930 birth cohort to 2.4 (95%CI 2.3 to 2.5) in the 1950–< 1960 birth cohort (Table 4). This decline was observed among women irrespective of race/ethnicity and country of birth (all p for trend < 0.001, *p* for interaction = 0.002). The proportion of women having fewer births (1–2 live births) increased from 32.3% (95%CI 29.3 to 35.5%) to 51.7% (95%CI 48.8 to 54.5) and who were grand multiparous decreased from (32.1% [95%CI 28.8 to 35.6%] to 19.9% [95%CI 17.6 to 22.5%]) (eTable 6). This pattern was observed irrespective of race/ethnicity or country of birth (*p* for interaction < 0.001). Except for Hispanic women (24.6% [95%CI 20.5 to 29.1%]), the proportion of grand multiparous women in the 1950–< 1960 birth cohort was < 20%.

**Table 4:** Weighted trends in the number of lifetime live birth overall, by race/ethnicity and country of birth among US women birth cohort 1910–< 1930 to 1950–< 1960^a^

	Trends in lifetime number of live birth (count, mean [SE]) birth cohort				P for trend^b^	Last vs. 1st birth cohort difference (95% CI)^c^
	1910–< 1930	1930–< 1940	1940–< 1950	1950–< 1960		
Overall	3.5	3.6	2.7	2.4	< 0.001	− 1.2
	(3.3 to 3.7)	(3.5 to 3.7)	(2.6 to 2.8)	(2.3 to 2.5)		(− 1.4 to − 1.0)
Race/ethnicity						
Hispanic	4.9	4.4	3.7	3.0	< 0.001	− 1.9
	(3.9 to 6.0)	(4.0 to 4.8)	(3.5 to 4.0)	(2.8 to 3.2)		(− 3.0 to − 0.8)
Non-Hispanic Black	3.8	4.3	3.1	2.6	< 0.001	− 1.2
	(3.4 to 4.2)	(3.9 to 4.7)	(2.9 to 3.2)	(2.4 to 2.8)		(− 1.7 to 0.8)
Non-Hispanic White	3.4	3.4	2.5	2.2	< 0.001	− 1.1
	(3.2 to 3.6)	(3.3 to 3.5)	(2.4 to 2.6)	(2.1 to 2.3)		(− 1.4 to − 0.9)
Remaining^d^	5.1	4.2	3.0	2.5	< 0.001	− 2.6
	(2.8 to 3.2)	(3.6 to 4.8)	(2.7 to 3.4)	(2.2 to 2.7)		(− 4.0 to − 1.2)
Country of birth						
US born	3.5	3.5	2.6	2.3	< 0.001	− 1.2
	(3.3 to 3.7)	(3.4 to 3.6)	(2.5 to 2.7)	(2.2 to 2.4)		(− 1.4 to 1.0)
Non-US born	4.1	3.9	3.3	2.8	< 0.001	− 1.3
	(3.5 to 4.7)	(3.6 to 4.2)	(3.0 to 3.5)	(2.6 to 3.0)		(− 1.9 to − 0.7)

^a^All data are weighted to be US nationally representative. Year of birth was derived as the lower bound of study cycle minus participants' age (i.e., the estimated year of birth for a participant aged 40 years in study cycle 1999–2000 would be 1959)

^b^P for trend were calculated using linear regression that included in the National Health and Nutrition Examination Survey (NHANES) estimated birth cohort as a continuous variable

^c^Indicates the absolute change in lifetime number of live birth between birth cohort 1910–< 1930 and 1950–< 1960, a decrease corresponds to lower number live birth over lifetime

^d^ “Remaining” includes race/ethnicity other than non-Hispanic white, non-Hispanic black, or Hispanic, including multiracial

Sensitivity analyses using the upper bound of study cycle to derive the year of birth yielded similar estimates (eTables 7–10).

## Discussion

In this nationally representative sample of US women, we observed a continued, substantial decline in age at menarche among all racial/ethnic groups comparing women born at the end of the twentieth century to those born at the beginning of the twentieth century. The decline was more rapid among women who were born outside the US. Among women who were born between 1990 and < 2000, 26.5% attained menarche before age 12, almost doubling the 14.2% for those born between 1910 and 1930. In addition, the proportion of women having their first live birth after age 30 doubled, reaching 10.6% among those born towards the end of the twentieth century. We observed no change in the age at natural menopause. However, reproductive pattern has changed with average lifetime live birth declining from 3.5 to 2.4 and an almost twofold reduction in the proportion of women who are grand multiparous.

Our findings are consistent with other studies. In the Apple Women’s Health study, a digital cohort of women who are Apple Research app and iPhone users, age at menarche declined from 12.5 years in their 1950–1969 birth cohort to 11.9 years in the 2000–2005 birth cohort [13]. A major difference is that while we analyzed data in a nationally representative sample, their study population is highly selective. Some of the earliest data documenting age at menarche at the population level are from Denmark and Norway and they have reported substantial declines from 17 years in 1840 to between 12.5 and 13 years in 2000. Other countries, including China and South Africa have also experienced rapid declines in age at menarche indicating this is a global phenomenon [16].

Our findings on delayed age at first live birth and decreased number of live births are also consistent with previous literature. Fertility rates increased in the 1940s and 1950s (baby boom), and then declined in the mid-1960s (baby bust) to 2 children per woman, similar to our estimates in 1910–< 1930 to 1950–< 1960 birth cohorts [17]. The fertility rates continued to decline to < 2 among women born between 1988 and 2010 [18–20]. Further, there is an increasing trend in US women giving births at a later age [19]. Meanwhile, there has been a decline in the number of live births globally, particularly grand multiparities, with fertility rates declining from 4.9/woman in the 1950’s to 2.3 in 2023 [21]. The most noticeable declines occurred in East/South/South East Asia, North Africa, Middle East, Latin America and Caribbean, where fertility rates are becoming comparable to those in high income countries [21].

An earlier age at menarche is associated with hormonal and metabolic dysregulation later in life activating the hypothalamic-pituitary-ovarian axis early, leading to extended lifetime exposure to steroid hormones, particularly estrogen [22]. Premenopausal women who experienced menarche early (< 12 vs. > 13 years) have higher early-follicular free estradiol (11%), estrone (7%), and free testosterone (8%) [23]. Further, a 1-year decrease in age at menarche is associated with an 8% increased risk in metabolic syndrome, independent of adult BMI [24]. Although these biological mechanisms influence incidence of breast cancer overall, impact on ER+ tumors appear to be stronger. Every one-year reduction in age at menarche increases overall breast cancer risk by 5%. Further, 9.8% of ER+ and 5.3% ER− breast cancers among postmenopausal women could be attributed to earlier age at menarche [25].

Parity modifies the associations of menarche with breast risk. A first live birth within 10 years of menarche reduces risk by 28% and each live birth by 16% [26]. However, late age at first birth (> 35 years) negates the protective effect of parity because the delayed mammary gland differentiation prolongs susceptibility to estrogen exposure [27]. Differentiated breast cells are more resistant to oncogenic transformation, and early full-term pregnancy and breastfeeding may reprogram breast tissue epigenetically, reducing the pool of vulnerable undifferentiated cells [28].

We observed no changes in the age at natural menopause suggesting that it is not likely to contribute to the rising breast cancer incidence. Rather, elevated and prolonged hormonal exposures earlier in life appear to be the main reproductive factors contributing. Reproductive factors likely exert their strongest biological effects during the premenopausal years, when ovarian function is active, and breast tissue is hormonally responsive [29]. However, the cancer-initiating processes set in motion by early-life exposures may not manifest clinically until postmenopausal years.

The dramatic changes in reproductive factors mirror both the US and global patterns in breast cancer incidence—increases in ER+ tumors, but decreases in ER− tumors [2, 30]. Approximately 10–15% of breast cancer cases are attributable to reproductive factors [31–33]. Although forecasting future trends in breast cancer incidence is beyond the scope of this study, our findings indicate that the current rise in breast cancer incidence is likely to continue. As more women transition towards lower parity and older ages at first birth, populations with low incidence rates of breast cancer will likely experience rapid increases in ER+ tumors, which account for > 80% of breast cancers. On the other hand, older age at first birth we observed may be contributing to the decrease in ER− tumors reported in recent literature [4]. These underscore the urgent need to adapt prevention strategies in response to the dynamic reproductive changes.

Changes in reproductive factors, especially earlier age at menarche, underscore the importance of early-life factors in breast cancer risk and the need for a comprehensive life-course approach to breast cancer prevention that starts early in life. The convergence in age at menarche for US and non-US born women also highlights the role of environmental factors in shaping age at menarche, with implications for breast cancer risk. Non-US born women arrive in the US with lower breast cancer risk but their risk over time converges with rates for US women, especially when they migrate to the US before age 20 [34, 35]. A shifting earlier age at menarche is likely to be of the factors contributing to the changing breast cancer risk profile in non-US born women. There is, thus, a need to better understand how factors influencing pubertal timing, such as exposure to endocrine disrupting chemicals and heavy metals, early life adversity and stress [36] impact breast cancer risk directly or indirectly through an earlier age at menarche and how these can be mitigated.

A key strength of the study is the comprehensive description of trends in reproductive factors among women across birth cohorts spanning nearly 100 years using nationally representative data. The serial cross-sectional design, although unable to track individuals over time, is particularly well suited for examining birth cohort effect at the population level. However, several limitations are noted. First, reproductive factors were self-reported. Nevertheless, self-reported reproductive factors have been widely used in epidemiologic studies, and measurement errors were unlikely to affect findings on trends across birth cohorts. Second, NHANES does not directly document participants’ year of birth, therefore, birth year was estimated using age and the lower bound of each study cycle. This approach may introduce misclassification particularly near cohort boundaries. Sensitivity analyses using the upper bound of study cycle, nevertheless, yielded similar findings. Third, we did not evaluate trends in breastfeeding, despite its protective effect on triple-negative breast cancer, because NHANES does not have consistent data on breastfeeding across birth cohorts.

## Conclusions

Over the past century, there has been a continued decline in mean age at menarche, number of births-per-woman and an increase in the proportion women having their first live birth after age 30. Our findings have wide ranging implications for breast cancer, particularly the rise in premenopausal ER+ breast cancers. As reproductive patterns that drive breast cancer incidence continue to change, prevention strategies must evolve to match these changes, especially in the younger generation.

## Supplementary Information

Below is the link to the electronic supplementary material.

Supplementary Material 1.

## Data Availability

The dataset generated and analyzed during the current study are publicly available and can be downloaded from the official NHANES website: https://wwwn.cdc.gov/nchs/nhanes/continuousnhanes/default.aspx

## Abbreviations

ER+: Estrogen receptor-positive
ER−: Estrogen receptor-negative
PR+: Progesterone receptor-positive
PR−: Progesterone receptor-negative
NHANSE: National Health and Nutrition Examination Survey
