# Supplementary material for: Generational trends in reproductive factors among women in the US: implications for breast cancer incidence
Source: Breast Cancer Res. 2026 Feb 3;28:54. doi: 10.1186/s13058-026-02222-x (PMC12954949; doi:10.1186/s13058-026-02222-x)
Supplement: Supplementary file 1 — Supplementary Material 1. [file 13058_2026_2222_MOESM1_ESM.docx]

**Supplementary materials**

**eTable 1. Sample size and weighted N among US women birth cohort 1910-<1930 to 1990-<2000**

**eTable 2. Weighted trends in prevalence of age at menarche <11, 12, 13 and 14 years among US women birth cohort 1910-<1930 to 1990**

**eTable 3. Weighted trends in mean age at natural menopause, overall and by race/ethnicity and country of birth among US women birth cohort 1910-<1930 to 1950-<1960**

**eTable 4. Weighted trends in mean reproductive lifespan, overall and by race/ethnicity and country of birth among US women birth cohort 1910-<1930 to 1950-<1960**

**eTable 5. Weighted trends in prevalence of women having a first live birth within 10 years of menarche, overall and race/ethnicity and country of birth among US women birth cohort 1910-<1930 to 1980-1990**

**eTable 6. Weighted trends in total number of lifetime live birth (percent) overall, by race/ethnicity and country of birth among US women birth cohort 1910-<1930 to 1950-<1960**

**eTable 7. Weighted trends in mean age at menarche, overall and by race/ethnicity and country of birth among US women birth cohort 1910-<1930 to 1990-<2000**

**eTable 8. Weighted trends in prevalence of age at menarche <11, 12, 13 and 14 years overall and age at menarche <12 years by race/ethnicity and country of birth among US women birth cohort 1910-<1930 to 1990-<2000**

**eTable 9. Weighted trends in prevalence of first live birth at age >30, overall and by race/ethnicity and country of birth among US women birth cohort 1910-<1930 to 1980-<1990**

**eTable 10. Weighted trends in the number of lifetime live birth overall, by race/ethnicity and country of birth among US women birth cohort 1910-<1930 to 1950-<1960.**

| **eTable 1. Sample size and weighted N among US women birth cohort 1910-<1930 to 1990-<2000^a^** | | | | | | | | | | | |
| --- | --- | --- | --- | --- | --- | --- | --- | --- | --- | --- | --- |
|  | **1910-<1930** | **1930-<1940** | **1940-<1950** | **1950-<1960** | **1960-<1970** | **1970-<1980** | **1980-<1990** | **1990-<2000** | **Total** | **Weighted%** |  |
| **Overall** | 1,581 | 2,861 | 3,482 | 4,062 | 4,307 | 4,408 | 5,317 | 2,463 | 28,481 | 100% |  |
|  | 4,014,946 | 8,535,724 | 12,641,447 | 17,741,367 | 18,744,258 | 16,658,386 | 15,123,733 | 7,342,310 | 100,802,171 |  |  |
| **Race/ethnicity** |  |  |  |  |  |  |  |  |  |  |  |
| **Hispanic** | 235 | 592 | 949 | 1,068 | 1,165 | 1,318 | 1,742 | 746 | 11,958 | 68.4% |  |
|  | 209,932 | 573,754 | 1,119,909 | 1,701,281 | 2,577,779 | 3,126,609 | 2,776,547 | 1,491,259 | 68,905,930 |  |  |
| **Non-Hispanic Black** | 194 | 499 | 738 | 1,060 | 975 | 886 | 1,332 | 633 | 6,317 | 11.8% |  |
|  | 281,007 | 729,790 | 1,171,029 | 2,092,121 | 2,329,245 | 2,195,413 | 1,996,217 | 1,091,797 | 11,886,618 |  |  |
| **Non-Hispanic White** | 1,120 | 1,668 | 1,553 | 1,601 | 1,778 | 1,767 | 1,733 | 738 | 7,815 | 13.5% |  |
|  | 3,437,982 | 6,962,436 | 9,618,405 | 12,927,798 | 12,679,783 | 10,111,593 | 9,054,094 | 4,113,840 | 13,577,071 |  |  |
| **Remaining^b^** | 32 | 102 | 242 | 333 | 389 | 437 | 510 | 346 | 2,391 | 6.4% |  |
|  | 86,025 | 269,744 | 732,104 | 1,020,167 | 1,157,451 | 1,224,771 | 1,296,875 | 645,414 | 6,432,551 |  |  |
| **Country of birth** |  |  |  |  |  |  |  |  |  |  |  |
| **US born** | 1,371 | 2,339 | 2,619 | 2,981 | 3,026 | 3,106 | 4,134 | 2,072 | 21,648 | 85.2% |  |
|  | 3,665,517 | 7,632,699 | 11,132,620 | 15,339,811 | 15,489,303 | 13,320,195 | 12,728,848 | 6,549,574 | 85,858,567 |  |  |
| **Non-US born** | 208 | 520 | 861 | 1,078 | 1,279 | 1,300 | 1,182 | 389 | 6,817 | 14.8% |  |
|  | 347,371 | 896,232 | 1,506,887 | 2,390,899 | 3,252,791 | 3,333,979 | 2,394,310 | 786,719 | 14,909,188 |  |  |
| a All data are weighted to be US nationally representative. Year of birth was derived as the lower bound of study cycle minus participants’ age (i.e., the estimated year of birth for a participant aged 40 years in study cycle 1999-2000 would be 1959).  b “Remaining” includes race/ethnicity other than non-Hispanic white, non-Hispanic black, or Hispanic, including multiracial. | | | | | | | | | | | |

| **eTable 2. Weighted trends in prevalence of age at menarche <11, 12, 13 and 14 years among US women birth cohort 1910-<1930 to 1990-<2000**^a^ | | | | | | | | | | | | | |
| --- | --- | --- | --- | --- | --- | --- | --- | --- | --- | --- | --- | --- | --- |
|  | **Trends in age at menarche (percentage [SE]) birth cohort** | | | | |  | |  | |  | | **P for trend^b^** | **Last vs. 1st birth cohort difference (95% CI)^c^** |
|  | **1910-<1930** | **1930-<1940** | **1940-<1950** | **1950-<1960** | **1960-<1970** | | **1970-<1980** | | **1980-<1990** | | **1990-<2000** |  |  |
| **<11 years** | **3.8** | **5.0** | **7.0** | **7.9** | **7.3** | | **9.8** | | **8.6** | | **10.5** | **<.001** | **6.7** |
|  | (2.0 to 5.0) | (4.2 to 6.0) | (5.9 to 8.2) | (6.9 to 9.1) | (6.3 to 8.4) | | (8.7 to 11.0) | | (7.7 to 9.6) | | (9.0 to 12.2) |  | (4.8 to 8.6) |
| **<12 years** | **14.2** | **16.5** | **21.7** | **20.5** | **19.7** | | **23.0** | | **23.4** | | **26.5** | **<.001** | **12.3** |
|  | (12.4 to 16.1) | (14.8 to 18.3) | (19.8 to 23.6) | (18.8 to 22.2) | (18.1 to 21.4) | | (21.5 to 24.5) | | (21.7 to 25.3) | | (24.3 to 28.9) |  | (9.3 to 15.3) |
| **<13 years** | **39.8** | **41.6** | **48.4** | **48.3** | **46.1** | | **50.9** | | **50.5** | | **52.9** | **<.001** | **13.1** |
|  | (37.6 to 42.0) | (39.3 to 43.9) | (46.1 to 50.7) | (46.3 to 50.2) | (44.0 to 48.2) | | (49.0 to 52.8) | | (48.5 to 52.5) | | (50.0 to 55.7) |  | (9.4 to 17.8) |
| **<14 years** | **66.1** | **71.5** | **75.2** | **74.0** | **72.6** | | **77.0** | | **76.0** | | **78.5** | **<0.01** | **12.3** |
|  | (63.4 to 68.8) | (69.3 to 73.6) | (73.2 to 77.1) | (72.3 to 75.7) | (70.7 to 74.5) | | (75.2 to 78.7) | | (74.2 to 77.6) | | (76.2 to 80.6) |  | (8.9 to 15.8) |
| a All data are weighted to be US nationally representative. Year of birth was derived as the lower bound of study cycle minus participants’ age (i.e., the estimated year of birth for a participant aged 40 years in study cycle 1999-2000 would be 1959)  b P for trend were calculated using linear regression that included in the National Health and Nutrition Examination Survey (NHANES) estimated birth cohort as a continuous variable.  c Indicates the absolute change in prevalence of age at menarche <11, 12, 13 and 14 years between birth cohort 1910-<1930 and 1990-2000, an increase corresponds to higher prevalence of younger age at menarche.  d “Remaining” includes race/ethnicity other than non-Hispanic white, non-Hispanic black, or Hispanic, including multiracial. | | | | | | | | | | | | | |

| **eTable 3. Weighted trends in mean age at natural menopause, overall and by race/ethnicity and country of birth among US women birth cohort 1910-<1930 to 1950-<1960**^a^ | | | | | | |
| --- | --- | --- | --- | --- | --- | --- |
|  | **Age at natural menopause (year, mean [SE]) birth cohort** | | | | **P for trend^b^** | **Last vs. 1st birth cohort difference (95% CI)^c^** |
|  | **1910-<1930** | **1930-<1940** | **1940-<1950** | **1950-<1960** |  |  |
| **Overall** | **49.2** | **50.1** | **50.4** | **49.4** | **0.565** | **0.2** |
|  | (48.8 to 49.6) | (49.8 to 50.3) | (50.2 to 50.6) | (49.1 to 49.7) |  | (-0.2 to 0.7) |
| **Race/ethnicity** |  |  |  |  |  |  |
| **Hispanic** | **48.6** | **48.8** | **49.3** | **48.7** | **0.777** | **0.1** |
|  | (47.1 to 50.1) | (48.2 to 49.4) | (48.8 to 49.7) | (48.2 to 49.1) |  | (-1.5 to 1.6) |
| **Non-Hispanic Black** | **49.5** | **49.9** | **50.2** | **48.7** | **0.003** | **-0.8** |
|  | (48.4 to 50.6) | (49.2 to 50.5) | (49.7 to 50.7) | (48.3 to 49.1) |  | (-1.9 to 0.4) |
| **Non-Hispanic White** | **49.2** | **50.2** | **50.6** | **49.6** | **0.701** | **0.4** |
|  | (48.8 to 49.6) | (49.8 to 50.5) | (50.3 to 50.9) | (49.2 to 50.0) |  | (-0.2 to 0.9) |
| **Remaining^d^** | **48.5** | **50.7** | **50.1** | **49.6** | **0.626** | **1.2** |
|  | (45.8 to 51.2) | (49.3 to 52.1) | (49.2 to 51.0) | (48.9 to 50.4) |  | (-1.6 to 4.0) |
| **Country of birth** |  |  |  |  |  |  |
| **US born** | **49.2** | **50.1** | **50.5** | **49.4** | **0.529** | **0.2** |
|  | (48.9 to 49.6) | (49.8 to 50.4) | (50.2 to 50.7) | (49.1 to 49.7) |  | (-0.3 to 0.7) |
| **Non-US born** | **49.0** | **49.7** | **49.9** | **49.4** | **0.901** | **0.5** |
|  | (47.7 to 50.2) | (49.1 to 50.3) | (49.5 to 50.4) | (49.0 to 49.9) |  | (-0.8 to 1.7) |
| a All data are weighted to be US nationally representative. Year of birth was derived as the lower bound of study cycle minus participants’ age (i.e., the estimated year of birth for a participant aged 40 years in study cycle 1999-2000 would be 1959).  b P for trend were calculated using linear regression that included in the National Health and Nutrition Examination Survey (NHANES) estimated birth cohort as a continuous variable.  c Indicates the absolute change in age at natural menopause between birth cohort 1910-<1930 and 1950-<1960, a decrease corresponds to younger age at natural menopause over lifetime.  d “Remaining” includes race/ethnicity other than non-Hispanic white, non-Hispanic black, or Hispanic, including multiracial. | | | | | | |

| **eTable 4. Weighted trends in mean reproductive lifespan, overall and by race/ethnicity and country of birth among US women birth cohort 1910-<1930 to 1950-<1960**^a^ | | | | | | |
| --- | --- | --- | --- | --- | --- | --- |
|  | **Mean reproductive lifespan (year, mean [SE]) birth cohort** | | | | **P for trend^b^** | **Last vs. 1st birth cohort difference (95% CI)^c^** |
|  | **1910-<1930** | **1930-<1940** | **1940-<1950** | **1950-<1960** |  |  |
| **Overall** | **36.2** | **37.1** | **37.7** | **36.8** | **0.337** | **0.5** |
|  | (35.9 to 36.6) | (36.8 to 37.4) | (37.4 to 38.0) | (36.5 to 37.1) |  | (0.1 to 1.0) |
| **Race/ethnicity** |  |  |  |  |  |  |
| **Hispanic** | **35.9** | **35.7** | **36.6** | **36.0** | **0.647** | **0.1** |
|  | (34.5 to 37.4) | (35.0 to 36.3) | (36.0 to 37.1) | (35.6 to 36.5) |  | (-1.5 to 1.7) |
| **Non-Hispanic Black** | **35.9** | **36.8** | **37.4** | **36.1** | **0.239** | **0.1** |
|  | (34.8 to 37.0) | (36.1 to 37.5) | (36.9 to 38.0) | (35.6 to 36.5) |  | (-1.0 to 1.3) |
| **Non-Hispanic White** | **36.3** | **37.3** | **37.9** | **37.0** | **0.137** | **0.7** |
|  | (36.0 to 36.7) | (37.0 to 37.6) | (37.6 to 38.3) | (36.6 to 37.4) |  | (0.1 to 1.2) |
| **Remaining^d^** | **33.9** | **37.2** | **36.7** | **36.6** | **0.492** | **2.7** |
|  | (31.5 to 36.3) | (35.3 to 39.0) | (35.7 to 37.8) | (35.9 to 37.4) |  | (0.1 to 5.4) |
| **Country of birth** |  |  |  |  |  |  |
| **US born** | **36.3** | **37.2** | **37.9** | **36.9** | **0.313** | **0.6** |
|  | (36.0 to 36.7) | (36.9 to 37.6) | (37.6 to 38.2) | (36.5 to 37.2) |  | (0.1 to 1.1) |
| **Non-US born** | **35.6** | **36.4** | **36.7** | **36.2** | **0.704** | **0.6** |
|  | (34.5 to 36.8) | (35.7 to 37.1) | (36.1 to 37.2) | (35.7 to 36.7) |  | (-0.7 to 1.9) |
| a All data are weighted to be US nationally representative. Year of birth was derived the lower bound of study cycle minus participants’ age (i.e., the estimated year of birth for a participant aged 40 years in study cycle 1999-2000 would be 1959).  b P for trend were calculated using linear regression that included in the National Health and Nutrition Examination Survey (NHANES) estimated birth cohort as a continuous variable.  c Indicates the absolute change in reproductive lifespan between birth cohort 1910-<1930 and 1950-<1960, a decrease corresponds to shorter reproductive lifespan over lifetime.  d “Remaining” includes race/ethnicity other than non-Hispanic white, non-Hispanic black, or Hispanic, including multiracial. | | | | | | |

| **eTable 5. Weighted trends in prevalence of women having a** **first live birth within 10 years of menarche, overall and race/ethnicity and country of birth among US women birth cohort 1910-<1930 to 1980-1990^a^** | | | | | | | | | |  |
| --- | --- | --- | --- | --- | --- | --- | --- | --- | --- | --- |
|  | **Age at menarche to first live birth within 10 years (percentage [SE])** | | | | | | | **P for trend^b^** | **Last vs. 1st birth cohort difference (95% CI)^c^** | |
|  | **1910-<1930** | **1930-<1940** | **1940-<1950** | **1950-<1960** | **1960-<1970** | **1970-<1980** | **1980-<1990** |  |  |  |
| **Overall** | **48.8** | **59.3** | **49.2** | **39.7** | **42.4** | **44.4** | **45.3** | **<.001** | **-3.5** | |
|  | (45.9 to 51.8) | (56.8 to 61.9) | (46.5 to 51.8) | (37.3 to 42.2) | (40.0 to 44.9) | (41.9 to 46.9) | (41.7 to 49.0) |  | (-8.1 to 1.1) | |
| **Race/ethnicity** |  |  |  |  |  |  |  |  |  | |
| **Hispanic** | **59.8** | **58.8** | **56.0** | **52.8** | **57.2** | **55.6** | **65.7** | **0.213** | **6.0** | |
|  | (46.2 to 72.0) | (52.7 to 64.6) | (50.9 to 60.9) | (48.1 to 57.6) | (53.7 to 60.6) | (51.8 to 59.3) | (59.9 to 71.1) |  | (-8.4 to 20.3) | |
| **Non-Hispanic Black** | **50.6** | **63.7** | **56.4** | **53.7** | **55.8** | **55.7** | **58.5** | **0.690** | **8.0** | |
|  | (42.1 to 59.0) | (58.9 to 68.2) | (52.0 to 60.7) | (50.0 to 57.3) | (51.8 to 59.8) | (51.2 to 60.1) | (52.5 to 64.3) |  | (-2.4 to 18.3) | |
| **Non-Hispanic White** | **48.2** | **59.0** | **47.1** | **35.9** | **37.4** | **39.2** | **35.5** | **<.001** | **-12.6** | |
|  | (44.8 to 51.5) | (55.9 to 62.2) | (43.8 to 50.5) | (32.6 to 39.3) | (34.3 to 40.7) | (35.8 to 42.8) | (30.2 to 41.3) |  | (-18.9 to -6.4) | |
| **Remaining^d^** | **42.3** | **56.1** | **51.9** | **34.5** | **34.0** | **31.0** | **32.4** | **<.001** | **-9.8** | |
|  | (24.5 to 62.3) | (45.9 to 65.8) | (43.2 to 60.4) | (27.4 to 42.3) | (27.1 to 41.6) | (23.8 to 39.3) | (23.8 to 42.5) |  | (-32.4 to 12.7) | |
| **Country of birth** |  |  |  |  |  |  |  |  |  | |
| **US born** | **49.1** | **60.4** | **49.4** | **39.3** | **41.7** | **44.5** | **43.5** | **<.001** | **-5.7** | |
|  | (46.0 to 52.3) | (57.6 to 63.2) | (46.5 to 52.3) | (36.6 to 42.1) | (38.9 to 44.5) | (41.6 to 47.5) | (39.1 to 47.9) |  | (-10.9 to -0.4) | |
| **Non-US born** | **45.6** | **50.6** | **47.6** | **42.2** | **45.9** | **44.0** | **52.1** | **0.800** | **6.5** | |
|  | (35.8 to 55.7) | (44.8 to 56.5) | (42.6 to 52.8) | (37.9 to 46.4) | (41.8 to 50.1) | (40.3 to 47.8) | (44.9 to 59.2) |  | (-5.9 to 18.9) | |
| a All data are weighted to be US nationally representative. Year of birth was derived the lower bound of study cycle minus participants’ age (i.e., the estimated year of birth for a participant aged 40 years in study cycle 1999-2000 would be 1959).  b P for trend were calculated using linear regression that included in the National Health and Nutrition Examination Survey (NHANES) estimated birth cohort as a continuous variable.  c Indicates the absolute change in reproductive lifespan between birth cohort 1910-<1930 and 1950-<1960, a decrease corresponds to shorter reproductive lifespan over lifetime.  d “Remaining” includes race/ethnicity other than non-Hispanic white, non-Hispanic black, or Hispanic, including multiracial. | | | | | | | | | |  |

| **eTable 6. Weighted trends in total number of lifetime live birth (percent) overall, by race/ethnicity and country of birth among US women birth cohort 1910-<1930 to 1950-<1960**^a^ | | | | |
| --- | --- | --- | --- | --- |
|  | **Lifetime total number of live birth (percentage [SE]) birth cohort** | | | |
|  | **1910-<1930** | **1930-<1940** | **1940-<1950** | **1950-<1960** |
| **Overall** |  |  |  |  |
| **0** | **1.8** | **0.7** | **1.5** | **1.6** |
|  | (1.2 to 2.9) | (0.4 to 1.2) | (0.9 to 2.5) | (1.0 to 2.7) |
| **1-2** | **32.3** | **28.1** | **47.5** | **51.7** |
|  | (29.3 to 35.5) | (25.8 to 30.4) | (44.3 to 50.7) | (48.8 to 54.5) |
| **3-4** | **33.8** | **39.3** | **29.8** | **26.8** |
|  | (30.9 to 36.8) | (36.8 to 41.7) | (27.5 to 32.3) | (24.4 to 29.3) |
| **5+** | **32.1** | **32.0** | **21.1** | **19.9** |
|  | (28.8 to 35.6) | (29.9 to 34.1) | (18.9 to 23.6) | (17.6 to 22.5) |
| **Hispanic** |  |  |  |  |
| **0** | **4.8** | **1.0** | **0.3** | **1.8** |
|  | (2.2 to 10.2) | (0.2 to 5.0) | (0.1 to 1.0) | (0.5 to 6.8) |
| **1-2** | **26.3** | **24.2** | **31.9** | **36.4** |
|  | (14.4 to 43.2) | (18.8 to 30.6) | (25.7 to 38.9) | (30.8 to 42.5) |
| **3-4** | **20.2** | **30.4** | **34.5** | **37.1** |
|  | (12.1 to 31.8) | (25.1 to 36.2) | (29.6 to 38.3) | (32.3 to 42.3) |
| **5+** | **48.7** | **44.4** | **33.2** | **24.6** |
|  | (34.9 to 62.7) | (37.4 to 51.6) | (28.3 to 38.4) | (20.5 to 29.1) |
| **Non-Hispanic Black** |  |  |  |  |
| **0** | **5.9** | **2.5** | **1.3** | **2.0** |
|  | (3.0 to 11.3) | (1.3 to 4.6) | (0.5 to 3.7) | (1.0 to 4.0) |
| **1-2** | **29.4** | **26.8** | **43.0** | **46.3** |
|  | (23.6 to 35.8) | (22.3 to 31.8) | (38.4 to 47.7) | (40.9 to 51.8) |
| **3-4** | **24.2** | **26.3** | **33.6** | **33.3** |
|  | (18.8 to 30.5) | (21.6 to 31.5) | (29.2 to 38.3) | (28.8 to 38.1) |
| **5+** | **40.6** | **44.5** | **22.0** | **18.3** |
|  | (33.9 to 47.7) | (38.8 to 50.4) | (18.4 to 26.0) | (14.8 to 22.5) |
| **Non-Hispanic White** |  |  |  |  |
| **0** | **1.3** | **0.6** | **1.6** | **1.6** |
|  | (0.7 to 2.6) | (0.3 to 1.1) | (0.9 to 2.8) | (0.9 to 3.1) |
| **1-2** | **33.4** | **29.0** | **51.0** | **54.6** |
|  | (29.7 to 37.2) | (26.4 to 31.7) | (47.3 to 54.7) | (50.8 to 58.3) |
| **3-4** | **35.9** | **41.9** | **28.5** | **24.2** |
|  | (32.5 to 39.5) | (39.1 to 44.7) | (25.6 to 31.6) | (21.1 to 27.5) |
| **5+** | **29.4** | **28.6** | **18.8** | **19.6** |
|  | (25.8 to 33.3) | (26.2 to 31.2) | (16.3 to 21.6) | (16.5 to 23.2) |
| **Remaining^b^** |  |  |  |  |
| **0** | **0.0** | **0.0** | **1.6** | **0.0** |
|  | NA | NA | (3.0 to 8.0) | NA |
| **1-2** | **19.8** | **21.0** | **35.0** | **54.7** |
|  | (7.9 to 41.6) | (13.7 to 30.9) | (26.2 to 45.0) | (45.2 to 63.9) |
| **3-4** | **23.6** | **32.0** | **33.4** | **26.9** |
|  | (12.4 to 40.3) | (21.8 to 44.2) | (25.4 to 42.4) | (19.3 to 36.0) |
| **5+** | **56.6** | **47.0** | **30.0** | **18.4** |
|  | (35.6 to 75.5) | (36.2 to 58.1) | (20.7 to 41.2) | (12.0 to 27.2) |
| **US born** |  |  |  |  |
| **0** | **1.8** | **0.8** | **1.6** | **1.8** |
|  | (1.0 to 3.0) | (0.4 to 1.3) | (1.0 to 2.8) | (1.0 to 3.1) |
| **1-2** | **32.8** | **27.2** | **48.6** | **53.2** |
|  | (29.2 to 36.5) | (24.8 to 29.8) | (45.1 to 52.2) | (50.0 to 56.3) |
| **3-4** | **34.1** | **40.8** | **29.6** | **25.3** |
|  | (30.8 to 37.6) | (38.1 to 43.6) | (27.0 to 32.4) | (22.7 to 28.1) |
| **5+** | **31.4** | **31.2** | **20.1** | **19.8** |
|  | (27.8 to 35.2) | (28.9 to 33.5) | (17.7 to 22.7) | (17.1 to 22.8) |
| **Non-US born** |  |  |  |  |
| **0** | **2.5** | **0.5** | **0.6** | **0.6** |
|  | (1.1 to 5.8) | (0.1 to 3.5) | (0.1 to 4.2) | (0.2 to 1.7) |
| **1-2** | **28.5** | **33.6** | **40.7** | **43.3** |
|  | (19.6 to 39.5) | (28.3 to 39.4) | (35.1 to 46.6) | (37.3 to 49.5) |
| **3-4** | **30.9** | **28.8** | **31.2** | **35.1** |
|  | (21.9 to 41.6) | (23.8 to 34.3) | (26.3 to 36.4) | (30.0 to 40.5) |
| **5+** | **38.1** | **37.1** | **27.5** | **21.0** |
|  | (30.0 to 47.0) | (31.4 to 43.1) | (22.6 to 33.0) | (17.2 to 25.3) |
| a All data are weighted to be US nationally representative. Year of birth was derived as the upper bound of study cycle minus participants’ age (i.e., the estimated year of birth for a participant aged 40 years in study cycle 1999-2000 would be 1960)  b “Remaining” includes race/ethnicity other than non-Hispanic white, non-Hispanic black, or Hispanic, including multiracial. | | | | |

| **eTable 7. Weighted trends in mean age at menarche, overall and by race/ethnicity and country of birth among US women birth cohort 1910-<1930 to 1990-<2000**^a^ | | | | | | | | | | |
| --- | --- | --- | --- | --- | --- | --- | --- | --- | --- | --- |
|  | **Trends in age at menarche (year, mean [SE]) birth cohort** | | | |  |  |  |  | **P for trend^b^** | **Last vs. 1st birth cohort difference (95% CI)^c^** |
|  | **1910-<1930** | **1930-<1940** | **1940-<1950** | **1950-<1960** | **1960-<1970** | **1970-<1980** | **1980-<1990** | **1990-<2000** |  |  |
| **Overall** | **13.0** | **12.9** | **12.6** | **12.6** | **12.7** | **12.5** | **12.5** | **12.5** | **<.001** | **-0.6** |
|  | (13.0 to 13.1) | (12.8 to 12.9) | 12.6 to 12.7) | (12.6 to 12.7) | (12.6 to 12.8) | (12.5 to 12.6) | (12.5 to 12.6) | (12.4 to 12.5) |  | (-0.7 to -0.5) |
| **Race/ethnicity** |  |  |  |  |  |  |  |  |  |  |
| **Hispanic** | **13.4** | **13.0** | **12.7** | **12.5** | **12.6** | **12.4** | **12.3** | **12.1** | **<.001** | **-1.2** |
|  | (13.0 to 13.8) | (12.9 to 13.2) | (12.5 to 12.9) | (12.3 to 12.6) | (12.4 to 12.7) | (12.3 to 12.5) | (12.2 to 12.4) | (12.0 to 12.3) |  | (-1.6 to -0.8) |
| **Non-Hispanic Black** | **13.1** | **13.0** | **12.7** | **12.6** | **12.6** | **12.3** | **12.2** | **12.3** | **<.001** | **-0.9** |
|  | (12.9 to 13.4) | (12.8 to 13.1) | (12.6 to 12.8) | (12.5 to 12.7) | (12.5 to 12.7) | (12.2 to 12.5) | (12.1 to 12.3) | (12.1 to 12.4) |  | (-1.2 to -0.7) |
| **Non-Hispanic White** | **13.0** | **12.8** | **12.6** | **12.6** | **12.7** | **12.6** | **12.6** | **12.7** | **0.001** | **-0.4** |
|  | (12.9 to 13.1) | (12.7 to 12.9) | (12.5 to 12.7) | (12.6 to 12.7) | (12.6 to 12.8) | (12.5 to 12.7) | (12.5 to 12.7) | (12.5 to 12.7) |  | (-0.6 to -0.3) |
| **Remaining^d^** | **13.5** | **13.4** | **13.1** | **13.2** | **12.9** | **12.7** | **12.6** | **12.4** | **<.001** | **-1.2** |
|  | (13.0 to 14.0) | (13.0 to 13.7) | (12.9 to 13.4) | (12.9 to 13.4) | (12.6 to 13.2) | (12.6 to 12.9) | (12.4 to 12.8) | (12.1 to 12.6) |  | (-1.7 to -0.6) |
| **Country of birth** |  |  |  |  |  |  |  |  |  |  |
| **US born** | **13.0** | **12.8** | **12.5** | **12.6** | **12.7** | **12.5** | **12.5** | **12.4** | **<.001** | **-0.6** |
|  | (12.9 to 13.1) | (12.7 to 12.9) | (12.5 to 12.6) | (12.5 to 12.7) | (12.6 to 12.7) | (12.4 to 12.6) | (12.4 to 12.5) | (12.4 to 12.5) |  | (-0.7 to -0.4) |
| **Non-US born** | **13.4** | **13.3** | **13.2** | **13.1** | **12.9** | **12.7** | **12.7** | **12.4** | **<.001** | **-1.1** |
|  | (13.1 to 13.7) | (13.2 to 13.5) | (13.0 to 13.4) | (12.9 to 13.2) | (12.8 to 13.0) | (12.6 to 12.8) | (12.6 to 12.8) | (12.2 to 12.6) |  | (-1.5 to -0.7) |
| a All data are weighted to be US nationally representative. Year of birth was derived as the upper bound of study cycle minus participants’ age (i.e., the estimated year of birth for a participant aged 40 years in study cycle 1999-2000 would be 1960)  b P for trend were calculated using linear regression that included in the National Health and Nutrition Examination Survey (NHANES) estimated birth cohort as a continuous variable.  c Indicates the absolute change in mean age at menarche between birth cohort 1910-<1930 and 1990-2000, a decrease corresponds to younger age at menarche.  d “Remaining” includes race/ethnicity other than non-Hispanic white, non-Hispanic black, or Hispanic, including multiracial. | | | | | | | | | | |

| **eTable 8. Weighted trends in prevalence of age at menarche <11, 12, 13 and 14 years overall and age at menarche <12 years by race/ethnicity and country of birth among US women birth cohort 1910-<1930 to 1990-<2000**^a^ | | | | | | | | | | | | | | | | | | | | |
| --- | --- | --- | --- | --- | --- | --- | --- | --- | --- | --- | --- | --- | --- | --- | --- | --- | --- | --- | --- | --- |
|  | | **Trends in age at menarche (percentage [SE]) birth cohort** | | | | | | | | |  | |  | |  | | **P for trend^b^** | | **Last vs. 1st birth cohort difference (95% CI)^c^** | |
|  | | **1910-<1930** | | **1930-<1940** | | **1940-<1950** | | **1950-<1960** | **1960-<1970** | | **1970-<1980** | | **1980-<1990** | | **1990-<2000** | |  |  |  |  |
| **<11 years** | | **3.7** | | **4.7** | | **6.9** | | **8.0** | **7.4** | | **9.3** | | **8.0** | | **10.0** | | **<.001** | | **6.3** | |
|  | | (2.7 to 5.2) | | (3.9 to 5.6) | | (5.8 to 8.2) | | (6.9 to 9.2) | (6.5 to 8.5) | | (8.3 to 10.4) | | (8.0 to 10.0) | | (8.6 to 11.7) | |  | | (4.4 to 8.2) | |
| **<12 years** | | **12.9** | | **16.1** | | **21.7** | | **20.5** | **20.3** | | **21.9** | | **24.2** | | **25.3** | | **<.001** | | **12.4** | |
|  | | (11.2 to 14.8) | | (14.5 to 18.0) | | (19.8 to 23.6) | | (18.9 to 22.1) | (18.8 to 21.9) | | (20.4 to 23.4) | | (22.2 to 25.8) | | (23.3 to 27.4) | |  | | (9.7 to 15.2) | |
| **Race/ethnicity** | |  | | | | | | | | | | | | | | | | | | |
| **Hispanic** | | **17.4** | | **16.7** | | **24.1** | | **28.8** | **22.8** | | **26.1** | | **30.6** | | **33.2** | | **<.001** | | **15.8** | |
|  | | (10.7 to 26.9) | | (12.2 to 22.3) | | (20.2 to 28.6) | | (24.7 to 33.3) | (19.6 to 26.5) | | (23.2 to 29.2) | | (27.7 to 33.6) | | (29..4 to 37.2) | |  | | (6.9 to 24.7) | |
| **Non-Hispanic Black** | | **13.9** | | **13.3** | | **21.2** | | **24.0** | **22.7** | | **29.1** | | **32.1** | | **27.9** | | **<.001** | | **13.0** | |
|  | | (9.4 to 20.0) | | (10.2 to 17.1) | | (18.4 to 24.4) | | (21.5 to 26.7) | (20.2 to 25.5) | | (25.9 to 32.5) | | (29.1 to 235.1) | | (24.4 to 31.7) | |  | | (7.5 to 20.6) | |
| **Non-Hispanic White** | | **12.8** | | **16.6** | | **21.9** | | **19.6** | **19.3** | | **19.7** | | **20.3** | | **21.3** | | **0.010** | | **8.5** | |
|  | | (10.9 to 14.9) | | (14.7 to 18.8) | | (19.6 to 24.4) | | (17.6 to 21.8) | (17.4 to 21.4) | | (17.5 to 22.1) | | (17.9 to 22.8) | | (18.5 to 24.4) | |  | | (4.9 to 12.1) | |
| **Remaining^d^** | | **3.6** | | **9.3** | | **14.9** | | **10.9** | **21.0** | | **16.7** | | **23.2** | | **29.8** | | **<.001** | | **26.2** | |
|  | | (0.5 to 22.1) | | (4.2 to 19.5) | | (10.0 to 21.7) | | (7.1 to 16.4) | (15.4 to 28.1) | | (!2.8 to 21.6) | | (18.1 to 29.2) | | (23.4 to 37.1) | |  | | (16.3 to 36.0) | |
| **Country of birth** | |  | | | | | | | | | | | | | | | | | | |
| **US born** | | **13.2** | | **16.7** | | **22.4** | | **21.1** | **21.0** | | **22.1** | | **24.5** | | **25.1** | | **<.001** | | **11.9** | |
|  | | (11.4 to 15.3) | | (14.8 to 18.7) | | (20.4 to 24.6) | | (19.3 to 23.0) | (19.2 to 22.9) | | (20.5 to 23.9) | | (22.5 to 26.7) | | (22.9 to 27.5) | |  | | (8.8 to 14.9) | |
| **Non-US born** | | **9.2** | | **11.2** | | **16.1** | | **16.5** | **16.9** | | **20.7** | | **21.0** | | **26.8** | | **<.001** | | **17.6** | |
|  | | (4.7 to 17.1) | | (8.3 to 15.0) | | (12.3 to 20.8) | | (13.5 to 20.0) | (14.3 to 19.9) | | (18.3 to 23.5) | | (18.0 to 24.4) | | (21.8 to 32.4) | |  | | (9.7 to 25.5) | |
| **<13 years** | | **39.0** | | **41.2** | | **48.8** | | **47.9** | **46.4** | | **49.8** | | **51.2** | | **52.1** | | **<.001** | | **13.1** | |
|  | | (36.5 to 41.6) | | (38.9 to 43.5) | | (46.5 to 51.2) | | (45.9 to 49.8) | (44.2 to 48.5) | | (47.8 to 51.8) | | (49.3 to 53.0) | | (49.4 to 54.9) | |  | | (9.3 to 16.9) | |
| **<14 years** | | **64.9** | | **71.2** | | **75.3** | | **74.2** | **72.7** | | **76.5** | | **76.1** | | **77.9** | | **<.001** | | **13.0** | |
|  | | (61.8 to 67.9) | | (69.1 to 73.1) | | (73.3 to 77.2) | | (72.3 to 75.9) | (70.8 to 74.4) | | (74.7 to 78.2) | | (74.4 to 77.7) | | (75.6 to 80.1) | |  | | (9.3 to 16.7) | |
| a All data are weighted to be US nationally representative. Year of birth was derived as the upper bound of study cycle minus participants’ age (i.e., the estimated year of birth for a participant aged 40 years in study cycle 1999-2000 would be 1960)  b P for trend were calculated using linear regression that included in the National Health and Nutrition Examination Survey (NHANES) estimated birth cohort as a continuous variable.  c Indicates the absolute change in prevalence of age at menarche <11, 12, 13 and 14 years between birth cohort 1910-<1930 and 1990-2000, an increase corresponds to higher prevalence of younger age at menarche.  d “Remaining” includes race/ethnicity other than non-Hispanic white, non-Hispanic black, or Hispanic, including multiracial. | | | | | | | | | | | | | | | | | | | | |
| **eTable 9. Weighted trends in prevalence of first live birth at age >30, overall and by race/ethnicity and country of birth among US women birth cohort 1910-<1930 to 1980-<1990**^a^ | | | | | | | | | | | | | | | | | | | |  |
|  | **Trends in first live birth age at >30 (percentage [SE]) birth cohort** | | | | | | | | | | | | |  | | **P for trend^b^** | | **Last vs. 1st birth cohort difference (95% CI)^c^** | |  |
|  | **1910-<1930** | | **1930-<1940** | | **1940-<1950** | | **1950-<1960** | | | **1960-<1970** | | **1970-<1980** | | **1980-<1990** | |  |  |  |  |  |
| **Overall** | **4.9** | | **2.8** | | **4.6** | | **8.2** | | | **10.2** | | **10.4** | | **9.3** | | **<.001** | | **4.4** | |  |
|  | (3.2 to 7.4) | | (2.1 to 3.8) | | (3.5 to 6.2) | | (6.7 to 9.9) | | | (8.6 to 12.1) | | (8.4 to 12.9) | | (6.6 to 12.8) | |  | | (0.7 to 8.1) | |  |
| **Race/ethnicity** |  | |  | |  | |  | | |  | |  | |  | |  | |  | |  |
| **Hispanic** | **9.5** | | **6.2** | | **3.6** | | **6.1** | | | **5.6** | | **5.0** | | **4.6** | | **0.645** | | **-5.0** | |  |
|  | (3.1 to 25.7) | | (3.1 to 11.9) | | (2.4 to 5.6) | | (3.7 to 9.9) | | | (4.1 to 7.5) | | (3.5 to 7.0) | | (2.5 to 8.3) | |  | | (-15.6 to 5.7) | |  |
| **Non-Hispanic Black** | **3.5** | | **1.5** | | **2.1** | | **3.4** | | | **5.9** | | **4.5** | | **3.7** | | **0.001** | | **0.2** | |  |
|  | (1.1 to 10.4) | | (0.7 to 3.4) | | (1.1 to 4.0) | | (2.1 to 5.3) | | | (4.4 to 7.9) | | (3.0 to 6.8) | | (1.7 to 7.9) | |  | | (-4.7 to 5.1) | |  |
| **Non-Hispanic White** | **4.5** | | **2.7** | | **5.1** | | **9.0** | | | **11.6** | | **13.3** | | **12.5** | | **<.001** | | **8.0** | |  |
|  | (2.8 to 7.2) | | (1.9 to 3.8) | | (3.6 to 7.2) | | (7.0 to 11.4) | | | (9.4 to 14.1) | | (9.9 to 17.5) | | (8.2 to 18.8) | |  | | (2.3 to 13.7) | |  |
| **Remaining^d^** | **11.6** | | **3.4** | | **4.5** | | **11.7** | | | **16.4** | | **14.8** | | **10.9** | | **0.002** | | **-0.7** | |  |
|  | (1.6 to 51.2) | | (0.5 to 19.4) | | (1.9 to 10.5) | | (7.2 to 18.5) | | | (10.1 to 25.4) | | (10.2 to 21.0) | | (5.3 to 21.2) | |  | | (-23.7 to 22.2) | |  |
| **Country of birth** |  | |  | |  | |  | | |  | |  | |  | |  | |  | |  |
| **US born** | **3.9** | | **2.3** | | **4.2** | | **7.7** | | | **10.3** | | **10.7** | | **9.7** | | **<.001** | | **5.9** | |  |
|  | (2.3 to 6.5) | | (1.6 to 3.3) | | (3.0 to 5.9) | | (6.2 to 9.7) | | | (8.5 to 12.4) | | (8.2 to 13.9) | | (6.6 to 14.1) | |  | | (1.6 to 10.1) | |  |
| **Non-US born** | **15.8** | | **7.0** | | **7.4** | | **10.1** | | | **10.1** | | **9.4** | | **7.8** | | **0.740** | | **-7.9** | |  |
|  | (8.6 to 27.1) | | (4.1 to 11.7) | | (4.3 to 12.4) | | (7.1 to 14.2) | | | (7.4 to 13.6) | | (7.0 to 12.4) | | (4.5 to 13.2) | |  | | (-18.0 to 2.1) | |  |
| a All data are weighted to be US nationally representative. Year of birth was derived as the upper bound of study cycle minus participants’ age (i.e., the estimated year of birth for a participant aged 40 years in study cycle 1999-2000 would be 1960)  b P for trend were calculated using linear regression that included in the National Health and Nutrition Examination Survey (NHANES) estimated birth cohort as a continuous variable.  c Indicates the absolute change in prevalence of first live birth over 30 years between birth cohort 1910-<1930 and 1980-<1990, an increase corresponds to higher prevalence of delayed first live birth.  d “Remaining” includes race/ethnicity other than non-Hispanic white, non-Hispanic black, or Hispanic, including multiracial. | | | | | | | | | | | | | | | | | | | |  |

| **eTable 10. Weighted trends in the number of lifetime live birth overall, by race/ethnicity and country of birth among US women birth cohort 1910-<1930 to 1950-<1960**^a^ | | | | | | |
| --- | --- | --- | --- | --- | --- | --- |
|  | **Trends in number of lifetime live birth (count, mean [SE]) birth cohort** | | | | **P for trend^b^** | **Last vs. 1st birth cohort difference (95% CI)^c^** |
|  | **1910-<1930** | **1930-<1940** | **1940-<1950** | **1950-<1960** |  |  |
| **Overall** | **3.5** | **3.6** | **2.8** | **2.4** | **<.001** | **-1.1** |
|  | (3.3 to 3.7) | (3.5 to 3.7) | (2.7 to 2.9) | (2.3 to 2.5) |  | (-1.3 to -0.9) |
| **Race/ethnicity** |  |  |  |  |  |  |
| **Hispanic** | **4.8** | **4.5** | **3.8** | **3.1** | **<.001** | **-1.7** |
|  | (3.5 to 6.2) | (4.1 to 4.7) | (3.5 to 4.1) | (2.9 to 3.3) |  | (--3.1 to -0.4) |
| **Non-Hispanic Black** | **3.8** | **4.4** | **3.1** | **2.6** | **<.001** | **-1.2** |
|  | (3.3 to 4.2) | (4.0 to 4.8) | (2.9 to 3.3) | (2.5 to 2.8) |  | (-1.6 to -0.7) |
| **Non-Hispanic White** | **3.3** | **3.4** | **2.6** | **2.2** | **<.001** | **-1.1** |
|  | (3.1 to 3.6) | (3.3 to 3.5) | (2.4 to 2.7) | (2.1 to 2.3) |  | (-1.4 to 0.9) |
| **Remaining^d^** | **5.0** | **4.5** | **3.1** | **2.5** | **<0.001** | **-2.5** |
|  | (3.5 to 6.5) | (3.9 to 5.1) | (2.7 to 3.4) | (2.2 to 2.8) |  | (-4.0 to -0.9) |
| **Country of birth** |  |  |  |  |  |  |
| **US born** | **3.5** | **3.6** | **2.7** | **2.3** | **<0.001** | **-1.2** |
|  | (3.2 to 3.7) | (3.5 to 3.7) | (2.6 to 2.8) | (2.2 to 2.4) |  | (-1.4 to -0.9) |
| **Non-US born** | **4.0** | **4.0** | **3.3** | **2.9** | **<0.001** | **-1.1** |
|  | (3.3 to 4.60) | (3.6 to 4.3) | (3.1 to 3.5) | (2.7 to 3.0) |  | (-1.8 to -0.4) |
| a All data are weighted to be US nationally representative. Year of birth was derived as the upper bound of study cycle minus participants’ age (i.e., the estimated year of birth for a participant aged 40 years in study cycle 1999-2000 would be 1960)  b P for trend were calculated using linear regression that included in the National Health and Nutrition Examination Survey (NHANES) estimated birth cohort as a continuous variable.  c Indicates the absolute change in lifetime number of live birth between birth cohort 1910-<1930 and 1950-<1960, a decrease corresponds to lower number live birth over lifetime.  d “Remaining” includes race/ethnicity other than non-Hispanic white, non-Hispanic black, or Hispanic, including multiracial. | | | | | | |
